# New international education project in predictive, preventive and personalized medicine

**DOI:** 10.1186/1878-5085-5-S1-A20

**Published:** 2014-02-11

**Authors:** Olga Safonicheva

**Affiliations:** 1 First Moscow State Medical University by I.M. Sechenov, Moscow, Russia

## Introduction

The concept of predictive personalized medicine is becoming one of the long-term priorities for the development of medical science in the structure of global trends. So, the main goal is: Development of educational programs and specialized didactic materials in PPPM for teachers of the universities and departments of post-graduate education, for students and for practical doctors. Program curriculum have to be structured according to the Bologna Process (European Higher Education Area). It is a difficult goal and we have to understand the new philosophy of up-to-date medicine for integration all the modern interdisciplinary innovations and the best researches of traditional eastern schools (Chinese and Ayurvedic). The duration of the Program maybe 2000 - 3000 academic hours (about 2-2, 5 years), it have to include several modules (4-5 modules need to be basic ones for doctors of all specializations).

## Module 1

Triad of health – mental, biochemical and structural components of phenotype. The study of health and life-quality biomarkers (“corridor of self-regulation”), markers of predisposition. Studying the status of healthy persons in order to understand their neuro-autonomic homeostasis, peculiarities of constitution and applied physiology. This module have to include express - diagnosis methods of visualization: optimal body posture, reflected zones of internal organs – “tissue substrates” in normal and in pathological condition; criteria of optimal cerebral metabolism, lymph circulation etc. New protocols.

## Module 2

Stress and General Adaptation Syndrome. Emotional, oxidative, muscle, postural stress - early preclinical diagnosis of stress-induced conditions (disturbed body statics, neuro-vascular “tunnel syndromes”, “endoecological crisis”) – non-specific grain for clinic behavior of multifactorial inflammatory and degenerative diseases.

## Module 3

Genetic testing of personal profiles: ONCO-gen, OSTEO-gen, CARDIO-gen, NEURO - gen, WEIGHT-gen etc. and correlation monitoring with clinical status in groups of risk. Learning the provoking role of stress-factors in the process of disease behavior. Epigenetic medical research - genetics of interaction, study of environmental and internal factors, ethics, culture and personal responsibility of an individual for his or her own health.

## Module 4

Creation of individual profiles in PPP programs, including conventional methods (modification of lifestyle, changing diet, physical activity) and medicines. Motivation and recommendations in PPPM for patients. Non-drug therapies for detoxification, correction of posture, cerebral metabolism, personalized exercises, intermitted hypoxia therapy etc.

## Conclusion

The education program needs to have 4-5 basic modules and every EPMA section may prepare special modules to accumulate the up-to-day knowledge and leading technologies with the purpose of creating interdisciplinary programs for maintaining the spiritual, psycho-emotional and physical health.

